# Variation in prescribing for anxiety and depression: a reflection of health inequalities, cultural differences or variations in access to care?

**DOI:** 10.1186/1475-9276-5-4

**Published:** 2006-05-18

**Authors:** Elizabeth Goyder, Chris Dibben, Michael Grimsley, Jean Peters, Lindsay Blank, Elizabeth Ellis

**Affiliations:** 1 School of Health and Related Research (ScHARR), University of Sheffield, Sheffield, UK; 2 School of Geography & Geosciences, University of St Andrews, St Andrews, UK; 3 School of Computing and Management Sciences, Sheffield Hallam University, Sheffield, UK

## Abstract

**Background:**

There are large variations in mental health prescribing in UK populations. However the underlying reasons for these differences, which may be related to differences in prevalence, cultural expectations or practical difficulties in access to treatment, remain uncertain.

**Methods:**

Linear modelling was used to investigate whether population characteristics or access to primary care account for variations in mental health prescribing across 39 deprived neighbourhoods.

**Results:**

The proportion of sampled respondents whose first language was not English and the ratio of general practitioners to population explained 61% of variation. Deprivation and mental health status were not significant predictors of prescribing in these relatively deprived communities.

**Conclusion:**

These findings suggest that mental health prescribing, within deprived areas, as well as reflecting cultural and social differences in prescribing, may also be a proxy measure of access to care.

## Introduction

There are large variations in drug prescribing in UK populations which are a cause of concern, particularly where they may reflect variations in the quality of care (whether through under or over prescribing relative to need). Previous studies have highlighted the wide variations in mental health related prescribing at practice level, [1,2] with Asian populations having consistently lower prescribing rates [3,4]. However the underlying reasons for these differences, which may be related to differences in prevalence, cultural expectations or practical difficulties in access to treatment, remain uncertain.

The aim of this study was to investigate whether variation in prescribing in deprived communities might be explained by underlying differences in access to primary care, as well as by characteristics of the practice and practice population.

## Methods

This work was undertaken as part of the establishment of baseline status of the 39 deprived areas participating in the New Deal for Communities (NDC) regeneration programme, funded by the Office of the Deputy Prime Minister (ODPM) and the Neighbourhood Renewal Unit (NRU). The NDC programme was created to address inequalities in these 39 deprived areas in England, each with a population of between 8000 and 12000 people.

The New Deal for Communities Household Survey was conducted by MORI (Market & Opinion Research International) and NOP (National Opinion Polls) as part of the national evaluation of the NDC programme. MORI and NOP interviewed approximately 500 residents aged 16 years and over from each of the 39 NDC areas between July and October 2002. Interviews were conducted face-to-face in the home, using Computer Assisted Personal Interviewing (CAPI). The sampling framework involved a random selection of addresses from within each of the NDC areas. The addresses were ordered by postcode, and 848 addresses were randomly selected in each (33,072 in total). No stratification was necessary given the relatively small size of the NDC areas. The interviewer randomly selected one property at each sampled address, one household within each selected property, and one adult within each selected household using a Kish grid. To be eligible, a person had to be aged 16 or over and normally resident at the address.

Detailed information was collected on population characteristics including mental health status. Data items collected included age, sex, ethnic group, first language, the mental health dimension of the SF36 [5] and perceived ease of access to general practice. For each NDC area, prescribing rates and the number of GPs per 10 000 population were calculated from data held by the Prescription Pricing Authority for 2002 and the Department of Health. In the calculation of prescribing rates, the numerator is the estimated number of 'days of treatment prescribed' based on the total amount of drugs prescribed and the "average daily quantity" (obtained from the NHS Information Centre [6]) which approximates the average daily dose. The denominator is the total number of patients on the practice list for April 2002. For each NDC, these rates were calculated for every practice covering the NDC population. A weighted average, based on the proportion of the NDC population served by each practice, was then calculated. Drug prescriptions were classified as anxiolytic drugs and anti-depressant drugs if included in BNF classification categories 4.1.2 and 4.3 respectively [7].

Potential explanatory variables considered were: self-reported ethnicity (proportion of the population defined as "Asian", "Black"and "White") and first language (proportion stating English was not their first language), proportion of the sampled population with poor self-reported health in the past year, area deprivation (Index of Multiple Deprivation 2001), mean respondent SF36 mental health score and both the sampled proportion with perceived "very/fairly easy" access to GPs and the number of GPs per 10 000 population (derived from data held by the Department of Health). Scatter plots of prescribing rates against each explanatory variable and linear regression (using SPSS for Windows version 10.0) were used to establish the association between explanatory variables and prescribing rates of anti-depressants and anxiolytics.

## Results

Prescribing rates varied widely between the 39 neighbourhoods (Table 1). In univariate analyses, only ethnicity, first language not being English and number of GPs were significant predictors of prescribing. In a multiple linear regression, including all the variables which were considered potential predictors of prescribing rates, only the proportion of the population not having English as a first language and the number of GPs per 10 000 population remained statistically significant independent predictors. A linear regression model including only these two variables predicted 61% of the observed variation. (Table 2). The multiple linear regression model including all the variables which were considered potential predictors (ie variables in Table 1) explained the variation less well than the model including only "English not the first language" and "number of GPs" (adjusted R^2 ^= 0.60). Similarly, a model including "Asian origin" and "number of GPs" but not "not having English as a first language" explained less of the observed variation (adjusted R^2 ^= 0.31).

**Table 1:** Population characteristics, GPs availability and prescribing in 39 neighbourhoods

**Explanatory variables**	Median	Range
*Ethnicity*		
"Asian" ethnic group (%)	4	0 to 57
First language other than English (%)	10	0 to 61
*Area Deprivation*		
Index of Multiple Deprivation (IMD 2000)	53.6	23.8 to 78.8
*Health*		
% "poor" health in past 12 months	23.5	14.5 to 33.3
Mental health SF-36 score (mean)	73.7	68.1 to 77.8
*Availability of primary care*		
% reporting "very/fairly easy access" to GP	71	56 to 87
Number GPs per 10 000 population	4.97	3.92 to 6.94

**Outcome: Prescribing rates **(average prescription days per head of population 2002)	Rate (interquartile range)	Range

Anxiolytic drugs (BNF category 4.1.2)	2.25 (1.51 to 3.10)	0.53 to 4.79
Antidepressant drugs (BNF category 4.3)	16.51 (12.16 to 16.51)	7.29 to 28.65
Anxiolytic + Antidepressant drugs	19.91 (13.77 to 24.23)	8.45 to 31.40

**Table 2:** Associations between population characteristics, availability of GPs and mental health related prescribing (anxiolytics + antidepressants) (n = 39)

**Explanatory variable**	**B (95% CI)**	**P value**	**Adjusted R**^**2**^
**Univariate linear regression**			

"Asian" ethnic group (%)	-0.22 (-0.33 to -0.10)	< 0.001	0.27
First language other than English (%)	-0.32 (-0.41 to -0.23)	< 0.001	0.56
Index of Multiple Deprivation (IMD)	-0.02 (-0.18 to 0.13)	0.8	0.03
% "poor" health in past 12 months	0.41 (-0.08 to 0.9)	0.10	0.05
Mental health SF-36 score (mean)	0.31 (-0.60 to 1.22)	0.5	0.01
% reporting "very/fairly easy access" to GP	0.19 (-0.10 to 0.48)	0.19	0.02
Number GPs per 100 000 population	3.14 (0.47 to5.82)	0.02	0.13

**Multivariate linear regression**^**a**^			

Number of GPs per 10 000 population	2.07 (0.28 to 3.87)	0.02	
First language other than English (%)	- 0.30 (-0.39 to -0.21)	< 0.001	0.61

^a ^Regression model including only "number of GPs per 10 000 population" and "first language other than English"

## Discussion

For these relatively deprived populations, prescribing for anxiety and depression are best predicted not by health status, material deprivation or ethnicity but by the proportion of the population with English as a first language and number of GPs per 10 000 population. These analyses suggest that the wide variation in prescribing rates observed in previous studies may be due not only to differences in health status or the cultural acceptability of treatment, but also to differences in access to primary care for those with mental health related symptoms, both in terms of the actual services available and an individual's ability to access them. It is worth noting that the number of GPs per 10 000 population was not correlated with area deprivation or self-reported health, variables that might predict increased need for primary care provision. As we did not have information about the prescribing practices, or any other characteristics, of GPs in the NDC areas, we cannot examine whether these associations are confounded by individual GP prescribing practices. For example, areas with larger Asian populations might also have more overseas trained doctors and this has previously been shown to be associated with lower prescribing rates independent of patient ethnicity[3]. Studies which explore the "supply side" factors that influence prescribing rates in more depth are needed to unravel these issues. Since the numbers of GPs in this data set was based on routine data which may not include accurate information on part-time GPs or GPs in training, some areas may have better provision than routine data suggests.

The lack of association with deprivation may well be due to all the areas identified as NDC communities being significantly deprived. A recent study from east London, that focused on practice characteristics, also found a correlation between anti-depressant prescribing and list size[4]. Similarly a lack of association with age and sex may be explained by the observation that these did not vary significantly between NDC areas.

The finding that the proportion of the population not having English as a first language was a significant predictor of prescribing rates, while proportion with "Asian" ethnicity was not, suggests a more complex relationship between culture, communication and prescribing, than can be measured by self-defined "Asian" ethnicity. It is plausible that identifying a language other than English as a first language reflects both language skills and self-perceived integration into the English-speaking community, both factors that may influence use of primary care services. The lack of independence of factors relating to ethnicity (particularly Asian ethnicity and English not as first language) means that it is difficult to be sure to what extent the underlying issue is language rather than ethnicity, but the multivariant analysis demonstrates that language is a better predictor than ethnicity and therefore potentially the underlying factor, reflected by ethnicity.

The major strength of this study was the availability of large sample survey data from the NDC MORI/NOP household survey. This gave us a validated measure of mental health status and allowed us to include mental health status as a potential explanatory variable. It also gave us a measure of perceived access as reported by respondents, although no information was collected on frequency of use of services. We had expected that at least some of the variation in prescribing between populations would be explained by underlying variation in the SF-36 mental health scores. The results suggest that for these communities, population need does not explain the variation. The remaining variation is likely to be largely due to random effects, local cultural attitudes to symptoms of anxiety and depression that are difficult to quantify and variation in the prescribing practice of individual GPs and other wider determinants of access to drug treatment.

Interpretation of prescribing rates need to bear in mind that these categories of drugs will be prescribed in a minority of cases for non-mental health conditions (for example neuropathic pain or enuresis), although it is likely that this would account for a relatively small proportion of variation. The clinical significance of variation in prescribing of anxiolytic and anti-depressant drugs may be different as there is some evidence from local audits that the former are over-prescribed and the latter under-prescribed relative to best practice. We found a high positive correlation between prescribing in these two categories, suggesting that in this analysis the prescribing patterns do not represent a proxy for "quality of care" (which might be predicted to show a negative correlation in that case) but purely for "access" irrespective of quality.

The main limitation in terms of the generalisability of our findings is that the data set only included relatively deprived urban populations that might be expected to have poorer than average access to GPs. It is possible that in more affluent populations with better access to primary care, population characteristics including mental health needs may explain some variation in prescribing rates. It seems likely, however, that if access is an issue, it will have an impact on prescribing.

These results do have important policy implications, particularly for those responsible for ensuring equitable and adequate access to primary care services. For example, when interpreting prescribing rates it may be worth considering that lower prescribing may not reflect better health or more judicious prescribing but instead reflect poorer access to care. Conversely, increasing prescribing rates may in fact be a reflection of improving access to care rather than deteriorating health status in a local population.

## Contributors

EG designed the study, analysed and interpreted the data, wrote the first draft of the paper and will act as guarantor for the paper. CD and MG participated in the data collection and analysis. All authors contributed to the original idea for the study and assisted with the writing of the paper.

## Funding

The collection of the data used in this paper was part of the National Evaluation of the New Deal for Communities (NDC) programme, funded by Neighbourhood Renewal Unit in the Office of the Deputy Prime Minister. However the content of this paper is the sole responsibility of the authors and does not reflect the views of the National Evaluation or of the Neighbourhood Renewal Unit in the Office of the Deputy Prime Minister.
